# Virtual Teaching Together: engaging parents and young children in STEM activities

**DOI:** 10.3389/fpsyg.2023.1334195

**Published:** 2024-02-06

**Authors:** Tricia A. Zucker, Michael P. Mesa, Michael A. Assel, Cheryl McCallum, Dana DeMaster

**Affiliations:** ^1^Children’s Learning Institute at University of Texas Health Science Center at Houston, Houston, TX, United States; ^2^University of Texas Health Science Center at Houston, Houston, TX, United States; ^3^Children’s Museum Houston, Houston, TX, United States

**Keywords:** outreach, learning, STEM, family engagement, preschool

## Abstract

**Introduction:**

Early informal learning experiences are essential for sparking long-term interest in science, technology, engineering, and math (STEM). In a prior study, we found more promising parent involvement outcomes when families of young children were provided with STEM family education events along with home STEM activity kits compared to providing workshops alone. This study was a conceptual replication using the same program—*Teaching Together STEM*—to deliver educational workshops plus home activity kits; however, we varied the delivery method by using virtual “funshops” to evaluate if parents perceived this modality as feasible and useful.

**Methods:**

Museum informal science educators introduced four units via virtual video chat sessions linked to 12 hands-on STEM activities that were mailed to families randomly assigned to the treatment group. Half of the families were assigned to a waitlist control group that received a portion of the virtual program after the posttest. Participants included 60 families with children aged 3 to 5 years from diverse linguistic and socioeconomic backgrounds.

**Results:**

Our results indicate no significant group differences in the primary outcome of parents’ involvement in informal STEM but a small, positive effect size (ES = 0.18) that was similar in magnitude to the prior, in-person study. Although parents mostly perceived the remote delivery as convenient and the materials as engaging for their child, there were no significant program impacts on children’s general science interests (ES = −0.19).

**Discussion:**

Despite the convenience, parents reported time was a barrier to doing STEM activities at home. Parents with lower education levels were less likely to attend, suggesting virtual approaches are not sufficient for ensuring broad access to family engagement programs for populations underrepresented in STEM.

## Introduction

This study examined the promise of reimaging a science, technology, engineering, and math (STEM) family engagement program with virtual facilitation. We designed a conceptual replication study that shifted from past in-person events to remote delivery of family education workshops called *Teaching Together STEM* (*TT STEM*). In this study and the past in-person version, the program was delivered by the same team of museum-based, informal STEM educators (hereinafter, “STEM educators”). We evaluated the promise of these virtual family “STEM funshops” using feasibility and usability outcomes and by comparing parent and child outcomes for the treatment group to a waitlist control group. This study occurred within 1 year into the global COVID-19 pandemic, and we designed the program for potential use beyond emergency contexts. If promising, virtual support for doing STEM at home might be part of our “new normal” post-COVID by providing unique spaces for families from diverse cultural and linguistic backgrounds to explore science and math with young children (Pattison et al., 2020; Zulirfan et al., 2020). Indeed, libraries and other community organizations increasingly offer virtual community engagement services that merit further evaluation (Evener and Chase, 2022).

### Broadening access to early STEM family engagement

Virtual family engagement approaches warrant study for two reasons. First, parents and caregivers report barriers to attending in-person family engagement events due to limited time or work and conflicting family schedules (Heath et al., 2018; Zucker et al., 2021, 2022). Nationally, only 6% of students have a parent attend school-based parent education workshops (National Center for Education Statistics, U.S. Department of Education, 2021). Virtual offerings could increase family attendance at educational events because it is a more convenient, flexible learning environment (Raes et al., 2020; Takeuchi et al., 2021) that could allow busy families with competing time priorities to do playful science activities when it best fits their schedule. However, there are potential challenges to virtual learning, such as reduced quality of interactions with the educator and other learners as well as potential for technology glitches or access issues (e.g., weak internet access speed; limited competencies for online platforms; Sullivan and Strawhacker, 2021).

Second, U.S. parents have less awareness of how to support their young child’s science and math skills compared to literacy (e.g., Sonnenschein et al., 2021). Yet, the U.S. needs to increase students’ general interest in STEM fields (National Research Council, 2009, 2012; Coley et al., 2020) to create pathways to long-term STEM interests and careers (Pattison et al., 2022). An important feature of early informal STEM experiences is to broaden access to address science and math learning opportunity gaps that begin early for students experiencing socioeconomic disadvantage as well as students who are Hispanic, Black, or American Indian (e.g., Morgan et al., 2016). Parents who speak languages other than English or parents with less formal education may particularly benefit from family engagement experiences that explain developmentally appropriate ways to get involved in their child’s learning and allow them to select their preferred language for STEM learning (Garibay, 2007; Green et al., 2007). Museum-based educators, librarians, and educators can host educational events to support parents of young children with messages, such as “science is for home, school and all the places in between…science is watchable, readable, playable and doable” (p. 52, Silander et al., 2018). Realizing how STEM is part of young children’s daily lives can empower parents to explore these concepts (e.g., Garibay, 2007; Šimunović and Babarović, 2020) and debunk common misunderstandings about who can do “real science” (e.g., Leblebicioglu et al., 2011).

### Rationale for our approach

Early science interest is important for developing a perception of yourself as someone who is capable of doing STEM (Kim et al., 2018; Lent et al., 2018; Archer et al., 2020). A major aim of our program was to increase children’s *interest in science*, which we conceptualized as a positive attitude, enjoyment, or value of doing science-related activities (Bell et al., 2019). Opportunities for increasing young children’s science interests are often playful and build off children’s questions about the world (e.g., Wolfgang et al., 2003; Casey et al., 2008; MacDonald et al., 2020).

Parents and caregivers play an important role in supporting children’s early science interests and STEM knowledge. The primary aim of our program was to increase *parent involvement in STEM* that includes home-based learning such as counting, comparing, talking about the natural world, and exploring STEM concepts that involve causal reasoning, problem-solving, or technical vocabulary (e.g., Haden et al., 2014; Silander et al., 2018; Cian et al., 2021). This was our primary outcome because parents of young children are the purveyors of many early STEM experiences and play key roles in shaping their children’s attitudes about STEM (e.g., Jacobs and Eccles, 2000). Parent involvement in learning activities is broadly related to student academic achievement (e.g., Sheldon and Epstein, 2005; Barnett et al., 2020; Ogg and Anthony, 2020). We aimed to increase parent involvement in STEM via a series of four virtual “funshops” and by mailing families hands-on STEM activities linked to the unit of study (Caniglia et al., 2021). We also sent parents follow-up text messages with tips and extension activities (Santana et al., 2019) and family museum passes (Vandermaas-Peeler et al., 2016; Pagano et al., 2020).

### Evidence for virtual learning

There are few rigorous experimental or mixed-method studies on the effectiveness of online learning for students in preschool to Grade 12 (Means et al., 2013; Poirier et al., 2019). To date, virtual or hybrid STEM research with young learners has mostly occurred in formal learning settings by integrating multimedia into classroom-based instruction (e.g., Rosenfeld et al., 2019). A few studies demonstrate the potential benefits of the virtual learning approach for preschool children and their parents in informal learning settings (e.g., McCarthy et al., 2013). Young children can gain knowledge from pre-recorded educational media that encourages extensions via social learning with caregivers (e.g., McCarthy et al., 2013; Rosenfeld et al., 2019; Neuman et al., 2020). Preschoolers can be as responsive to conversations through video chat platforms like Zoom as they are to in-person conversations; they also have similar vocabulary and comprehension benefits via video chat compared to in-person modalities (Gaudreau et al., 2020). For parents, there is some evidence that their attitudes and abilities to support their child’s learning improve after participating in virtual learning programs (e.g., Pasnik et al., 2015). Thus, there is initial evidence that virtual approaches to engaging children and families in STEM warrant further research using rigorous experiments and implementation science lenses that consider outcomes, such as feasibility and usability (e.g., Proctor et al., 2011; Atkins et al., 2017).

### Current study

The *TT STEM* program is designed for 3- to 5-year-old children to explore science, math, and engineering concepts with support from a parent or caregiver (hereinafter, referred to as parents, given that was the majority of our sample). We modified the existing, in-person *TT STEM* “funshops” due to COVID-19, but we hoped this virtual approach might prove useful post-pandemic. This was a conceptual replication study because we hypothesized that the virtual version of the TT STEM program could produce small increases in parent involvement in science and math commensurate in magnitude with effect sizes [ES] observed in an earlier, in-person study (ES range = −0.08 to 0.18; Zucker et al., 2022). Both the prior study and the current study used very similar materials and methods, such as the same STEM educators as funshop facilitators and a series of follow-up text message reminders and extension activities after each event. We primarily compared the virtual treatment group of this study to a waitlist control group of families; moreover, we also compared the magnitude of effect sizes in this virtual study to the prior in-person version of the program. Our recruitment approach included both schools that were partners in the first study and social media; this resulted in a sample of families from diverse socioeconomic, racial, and linguistic backgrounds. We expected linguistic diversity and, thus, offered a bilingual program with a choice of English or Spanish virtual sessions and text messages. We used an experimental design and mixed method data sources to understand if this virtual approach improved key parent and child outcomes and was feasible for families to take part in. We addressed the following three research questions (RQ):

1. To what extent was the virtual treatment feasible and useable in terms of parent perceptions, session attendance, activity utilization, and overcoming parents’ perceived barriers to doing STEM at home?
2. Did the program impact parent involvement in informal STEM learning?
3. Did the program impact children’s science interests?

For the first set of implementation outcomes, we expected variability in parent attendance but that the virtual program would reduce barriers to doing STEM at home. In regard to measures, we hypothesized the parent involvement survey that assessed several ways of doing science and math within the family’s daily routines to be appropriate to detect effects. We also gathered qualitative data describing how parents supported their child’s learning in ways that fit their unique family context. We were not certain if the rather generalized child interest survey would be sensitive enough to detect changes; however, it aligned with our logic model and other similar approaches that theorize early family participation in informal STEM can promote long-term STEM interest (e.g., Pattison et al., 2022).

## Methods and materials

This study was conducted in 2021—as the COVID-19 pandemic was ongoing—by university-based researchers and museum-based STEM educators in a research–practice partnership. Participants included 3- to 5-year-old children and their parents. We recruited via school-based flyers and online/social media advertising (i.e., Museum and University’s social media and newsletters). Most families resided in an urban U.S. city where the Children’s Museum Houston is located; however, a few were recruited via social media from rural areas in this U.S. state. We recruited 60 families and randomly assigned 30 to waitlist control and 30 to treatment. As detailed in Table 1, approximately, 48% of the children in the sample were girls (*M*_age_ = 4.67 years, SD = 0.57), and 50% of families spoke a language other than English. Among these families, nine selected Spanish as their preferred language of communication. For ethnicity, 42% reported that their child was Hispanic. In terms of child’s race, the sample was 63% white, 28% African American, 13% Asian, and 5% other. Mothers’ median education was a master’s or postgraduate degree, and fathers’ median education was a bachelor’s degree. Median household income was $40,001–$70,000 (missing data for *n* = 12) with a sizeable range from ≤$11,000 to ≥$150,000. Approximately 38 and 55% of mothers and fathers reported a STEM-related career, respectively. Children participated in two formal education settings: 30 different early childhood centers (90% of the sample) and homeschooling (10%). Parents/primary caregivers provided written informed consent (Study #HSC-MS-15-0759) prior to their inclusion in the study. We randomized families without accounting for baseline demographics; however, language preference was relatively balanced across conditions, with five Spanish-speaking families assigned to the treatment group and four Spanish-speaking families in the control group.

**Table 1:** Participant baseline demographic characteristics and balance check for posttest analytic sample (*n* = 50); means and (standard deviations).

	Treatment (*n* = 23)	Control (*n* = 27)	Unstandardized regression coefficient (attriters—non-attriters)	Difference as effect size	*p*-value
Demographic and family characteristics					
Child female?	0.43 (0.51)	0.48 (0.51)	−0.05	−0.09	0.747
Other language at home?	0.48 (0.51)	0.56 (0.51)	−0.08	−0.15	0.595
Mother’s education^a^	6.78 (2.43)	6.70 (2.71)	0.08	0.03	0.915
Father’s education^a^	5.87 (2.90)	5.30 (3.07)	0.57	0.19	0.503
Mother STEM-related career	0.30 (0.47)	0.37 (0.49)	−0.07	−0.13	0.632
Father STEM-related career	0.57 (0.51)	0.53 (0.49)	−0.06	−0.13	0.651
Is child hispanic?	0.45 (0.51)	0.41 (0.50)	0.05	0.09	0.746
Child’s race					
Black/African American	0.30 (0.47)	0.19 (0.40)	0.12	0.30	0.335
White	0.70 (0.47)	0.63 (0.49)	0.07	0.13	0.632
Asian	0.09 (0.28)	0.22 (0.42)	−0.14	−0.32	0.201
Other	0.00 (0.00)	0.07 (0.27)	−0.07	−0.28	0.190
Household income^b^	4.71 (2.05)	5.59 (2.03)	−0.89	−0.43	0.189
Baseline outcome measures					
Parent involvement^c^	2.65 (0.53)	2.49 (0.50)	0.16	0.32	0.283
Child STEM Interest^d^	3.53 (0.43)	3.44 (0.45)	0.09	0.20	0.481

a Education was measured as an 8-category variable ranging from 1 to 8, where 1 represents < =8th grade and 10 = master or postgraduate degree.

b Household income was measured as an 8-category variable ranging from 1 (11 K or less) to 8 ($150 K or more).

c Ranges from 1 = none to 4 everyday.

d Ranges from 1 = strongly disagree to 4 = strongly agree.

Overall F-test for the posttest sample, where all variables listed in the table were used to predict attrition, was statistically significant, F (18, 20) = 1.37, *p* = 0.247.

### Treatment procedures

The virtual *TT STEM* funshops were delivered across 10 weeks (March–May 2021) and addressed four units detailed in Supplementary Table S1. Two bilingual (English/Spanish) Hispanic female STEM educators with 11 and 19 years of experience in family engagement delivered sessions. Five treatment families (16.7%) selected the Spanish version of the sessions and text messages. Table 2 shows screenshots of key steps from unit 1. Each unit included the following five procedures:

1. **Mailed activity kit**: the museum educators mailed families a kit of three activities about one week before each unit introduction chat was scheduled (12 total activities).
2. **Introductory chat**: in a 20-min synchronous video chat, STEM educators used an icebreaker activity to generate excitement for the “funshop” thematic unit. Next, the facilitator introduced the unit topic and kit activities. Families used their own devices to join a Zoom meeting in their preferred language (English-4:30 pm or 5:30 pm; Spanish-4:30 pm).
3. **Home activities**: families were sent English or Spanish text messages with a link to YouTube channels created by the museum and designed for parent–child co-viewing to include the following: (a) unit introduction, (b) STEM educators read-aloud modeling focal parent strategies, and (c) three videos with instructions/models for each of three kit activities. Each activity included bilingual step-by-step instructions with photos to minimize reading demands.
4. **Follow-up chat**: approximately 2 weeks later, in a 20-min Zoom follow-up discussion, families were encouraged to share artifacts from their completed projects and discuss what they remembered or learned with activities.
5. **Extensions**: parents received text messages with tips to continue supporting their child’s STEM learning to use the strategies modeled by the STEM educators in extension activities linked to the theme but using common household objects (see Table 2).

**Table 2:** Virtual teaching together STEM cycle of activities.

Modality	Activity description	Screenshot/Photos
N/A	**Preparation**—mail kits with links to Zoom meeting times/details. Send text reminders. Login to Zoom meeting.	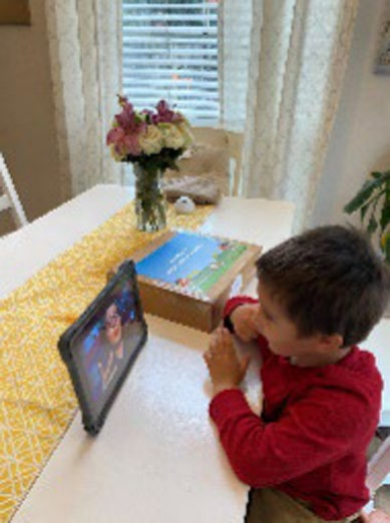
Synchronous	**Intro video chat**—welcome and preview activities and parent strategies (15–20 min). Every 2 weeks, a new unit was introduced with hands-on icebreaker activity ISE guided families to complete together.	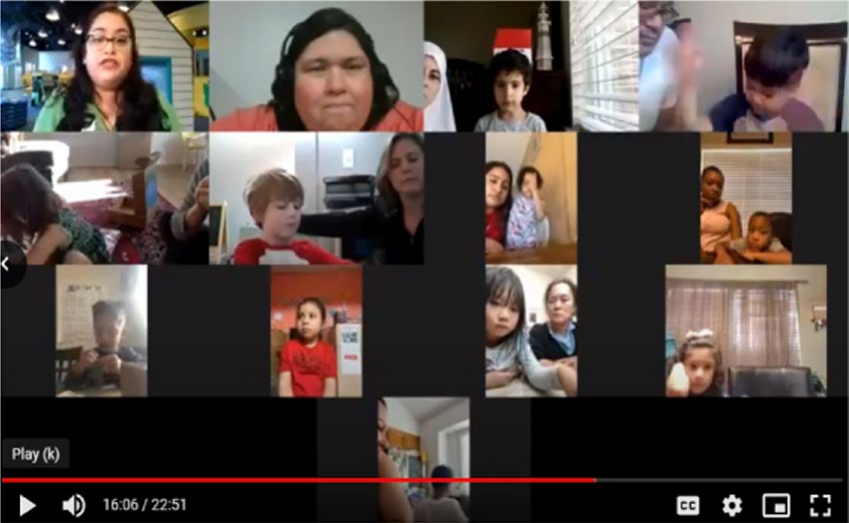
Asynchronous	**Unit kickoff video**—explains how to do science and math with young children. Explains parent strategies of using big words and asking open-ended questions	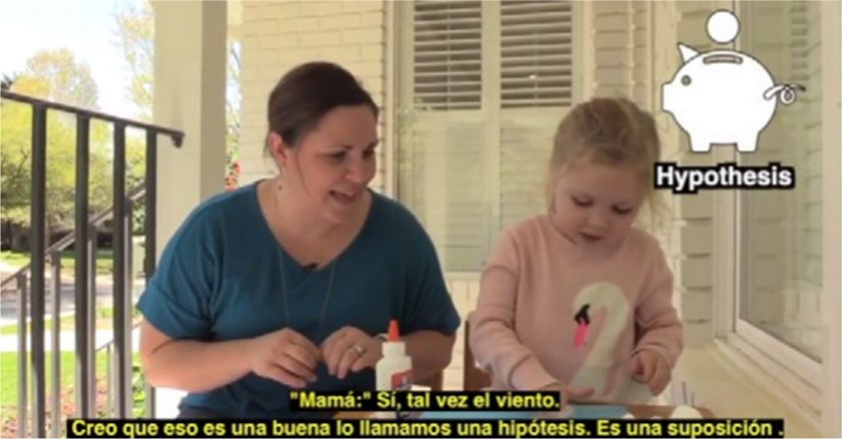
	**Read aloud video**—informal STEM expert from museum model strategies during read-aloud of a text	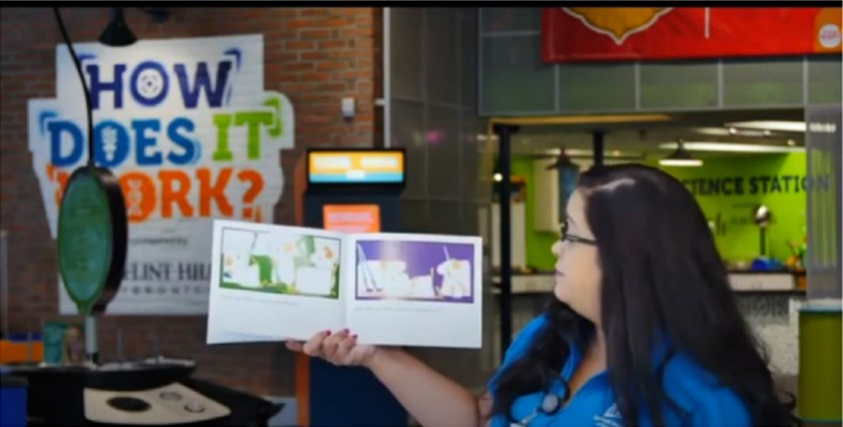
	**Activity preview videos**—Informal STEM expert from the museum explains each of the three activities in the family’s mailed kit	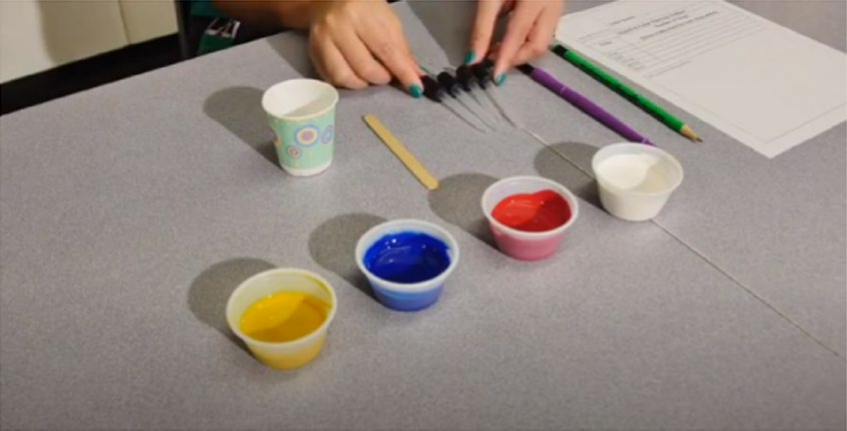
Asynchronous^a^	**Parent–child home activities**—Family completes the STEM activities in their mailed kit using detailed instructions from ISE with linked bilingual YouTube video demonstrations	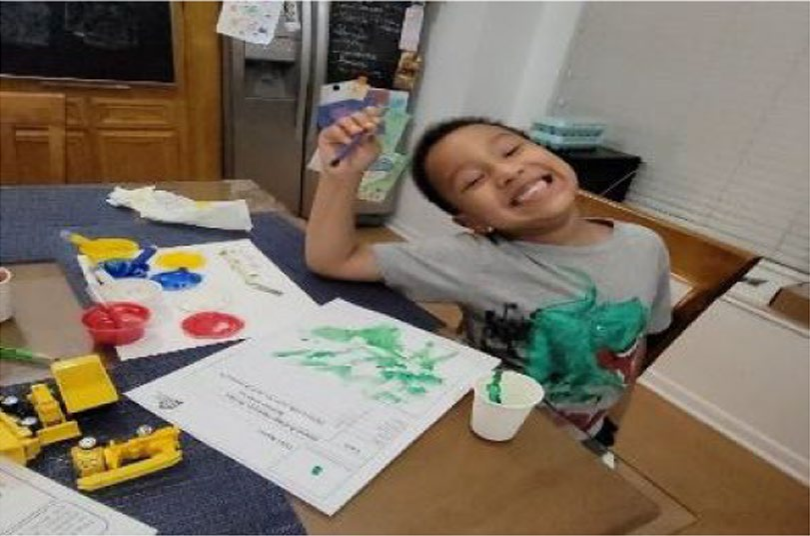
Synchronous	**Follow-up video chat**—Show and tell about STEM activity/creations and request to complete a feedback survey (20–25 min)	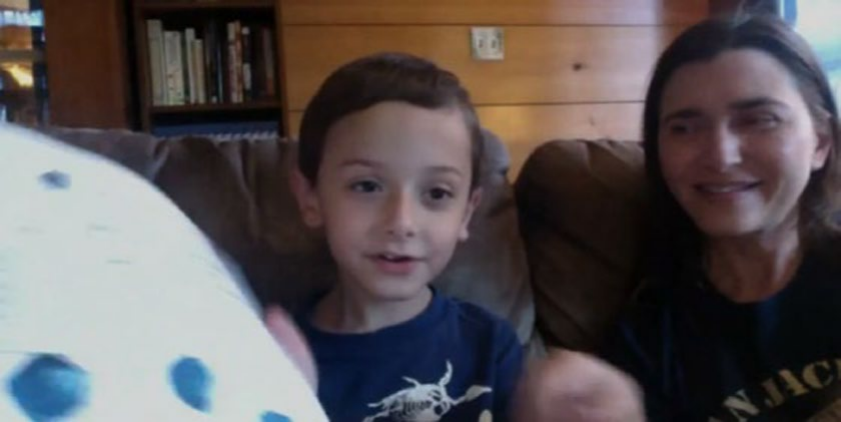
Asynchronous	**Extensions**—Parents receive text messages with tips and extension activities to further increase parent involvement in STEM activities	

a Parents were sent a QR code and a link with video instructions that were in their preferred language. Theme 1 instructional videos are available at this YouTube channel in English https://www.youtube.com/playlist?list=PLPZCH1CZOF9IJPQOxPJ0Xp0fkp8egc_NG and here in Spanish https://www.youtube.com/playlist?list=PLPZCH1CZOF9JhZI7gtiCwYEvgXAJBakzZ.

At the conclusion, participants received a family pass ($72 value) for museum entry, when it reopened in June 2021 after COVID-related gallery closures. The logic model for this treatment approach is illustrated in Supplementary Figure S1. The materials mailed to families were exactly the same as the in-person funshop materials mentioned in our study, but we selected a portion of past materials because we only delivered four of the six available workshop themes in this brief virtual intervention. We also did not send the nine supplemental materials given in two groups of our prior study (B and C; Zucker et al., 2022) because that would have resulted in a likely overwhelming number of activities for parents to use and because the cost of mailing these exceeded typical museum outreach budgets. The text messages and facilitators in modeling videos were exactly the same as in our initial study.

### Waitlist control procedures

The waitlist control group received one unit on a delayed schedule *after* the posttest (i.e., the posttest was completed by June, and the virtual waitlist program was offered in July 2021). STEM educators delivered the first unit only (What’s the Big Idea) using procedures #1–4 mentioned above. That is, families were mailed one kit and took part in the video chats with a STEM educator. Limited resources prevented us from offering the full series of virtual themes. Waitlist control families did not receive text messages, as this followed an automated schedule that matched the larger intervention delivery schedule.

### Measures

Given that this was a pilot, we used a brief online parent survey to capture only a small number of key outcomes. The pretest occurred from mid-January to February 2021, and the posttest was in June 2021. Upon completion, families received a $25 eGift card. Table 3 reports descriptive statistics and missing data details. The primary outcome was a quantitative measure of parents’ home-based involvement. *Parent involvement in STEM* was measured with nine items about the frequency of STEM-related activities. Items were adapted from the Head Start Family and Child Experiences Survey (FACES; West et al., 2007), such as: “How many times in the past week have you compared sizes of objects or toys with your child?” “How many times in the past week have you talked to your child about plants, animals, or other living things?” These were the same items used in our past study (Zucker et al., 2022).

**Table 3:** Descriptive statistics for outcomes for analytic sample (*n* = 50); means and (standard deviations).

	Treatment (*n* = 23)		Control (*n* = 27)	
	Pretest *M* (SD)	Posttest *M* (SD)	Pretest *M* (SD)	Posttest *M* (SD)
Parent involvement^a^	2.65 (0.53)	2.73 (0.45)	2.49 (0.50)	2.57 (0.69)
Child STEM interest^b^	3.53 (0.43)	3.64 (0.47)	3.44 (0.45)	3.60 (0.43)

a Range from 1 = none to 4 everyday.

b Range from 1 = strongly disagree to 4 = strongly agree.

We also gathered qualitative data related to how parents supported their child’s STEM learning. During the program, we asked treatment and control parents to send us a short text message in response to this prompt: “Tell us about an activity you did with [child name customized here] this week to support his/her learning.” We requested three texts from treatment parents and received 24 written replies (26.7% response rate) and two times from control with 18 replies (30.0% response rate). With the treatment group only, we also used an exit survey after each theme. The exit survey asked about which read-alouds and provided STEM activity kits they used as well as their parent involvement goals (“How do you plan to support your child’s learning?”). This exit survey was accessible at the end of the video chat with QR codes and was also sent to all treatment parents with links to text messages scheduled after the follow-up sessions. We had 41 qualitative responses across all four exit surveys, resulting in a relatively low response rate of 46.0%. At posttest only, we had a secondary, qualitative measure tapping STEM barriers (“What do you think are the top barriers to families doing science and math activities at home?”).

The primary child outcome was a general interest in science, as rated by their parent. We adapted items from the *Student Interest in Technology and Science* (SITS; Romine et al., 2014). This included three items about learning (“My child enjoys learning science”; “My child likes it when we find ways to do science outside of school”) and one career item (“I think my child would like to work in a science-related career one day”) on a 4-point scale from *1* = strongly disagree to *4* = strongly agree. Internal validity for this sample was α = 0.80. A secondary measure for treatment families was children’s interest in the virtual *TT STEM* program activities. We asked families about each unit’s three activities (12 total) and how interested their child was in these individual activities with a 5-point scale (*1* = extremely interested, *5* = not at all interested). These child interest measures were not used in our initial study but were added in this replication to assess more aspects of our logic model.

### Data analysis

The analysis plan was pre-registered using the Registry of Efficacy and Effectiveness Studies (Registry ID: 9800.1v1); however, we deviated from the original pre-registration plan that had expected primarily school-based recruitment; however, adding school-level fixed effects was not appropriate, so we dropped that model. We estimated the intent-to-treat (ITT) of being assigned to participate in treatment using OLS regression, using the equations below, where *Y* is the parent or child-level outcome, *i* denotes child or parent, and *s* denotes school. We included the pretest score *β_2s_* and child-level covariates *β_3s_*:


[Model 1] $$Y_{\mathrm{is}}=\beta_{0s}+\beta_{1}\left({\mathrm{Treatment}}_{s}\right)+\beta_{2s}\;\left({\mathrm{Pretest}}_{\mathrm{is}}\right)+\varepsilon_{\mathrm{is}}$$

[Model 2] $$\begin{array}{l} Y_{\mathrm{is}}=\beta_{0s}+\beta_{1}\left({\mathrm{Treatment}}_{s}\right)+\beta_{2s}\;\left({\mathrm{Pretest}}_{\mathrm{is}}\right)+\\ \beta_{3s}\;\left({\mathrm{Covariate}}_{\mathrm{is}}\right)+\varepsilon_{\mathrm{is}} \end{array}$$

Model 1 adjusts for pretest scores and basic controls; Model 2 adds adjustments for child demographics. To examine qualitative data, the lead and second author reviewed transcripts of verbatim responses and coded them using implementation science domains (Atkins et al., 2017). We calculated inter-rater agreement (92%) and reached a consensus on conclusions.

## Results

We detail participation in study activities and attrition in Supplementary Tables S3 and S4. These tables show no significant baseline group differences but marginal trends. Attrition was higher in the treatment than in the control group. Treatment parents were more likely to attrite at posttest if they had lower education levels.

### RQ1: treatment feasibility

#### Perceived satisfaction

Parents were assigned to treatment completed satisfaction surveys using a Likert scale (e.g., *1* = very useful, *5 =* not useful; e.g., “How helpful were the YouTube funshop videos in helping you and your family learn new ways of doing science and math at home?”). Ratings of satisfaction immediately after funshops (*n* = 41 responses) suggest good approval, *M* = 1.79 (SD = 0.98). At posttest, over 90% of parents (*n* = 20 respondents) said that the *TT STEM* program was helpful: (a) initial, synchronous video sessions, *M* = 1.32 (SD = 0.58); (b) YouTube videos explaining activity kits, *M* = 1.26 (SD = 0.45); (c) follow-up, show-and-tell video sessions, *M* = 1.32 (SD = 0.48); (d) text messages with parent tips, *M* = 1.35 (SD = 0.59); and (e) text message extension activities, *M* = 1.35 (SD = 0.49). Parents’ open-ended responses indicated key benefits were convenience and the ability to select English or Spanish sessions. Similarly, interviews with STEM educators indicated they would like to “maintain virtual and in-person formats so families can choose what works better for them…we are facing a new era where technology is the ‘main character”. So, we need to offer virtual sessions – not just for an emergency.” Both STEM educators reported greater self-efficacy for facilitating in-person family events than virtual family events. For example, one ISE explained “sometimes we missed the fun” during virtual events. She elaborated, “There’s a difference between the excitement in person—when they walk in the room, they are already excited. They see all the activities…they are like ‘wow!’ They cannot wait to try them out. But in virtual, I feel like they still were able to get excited because they would get this beautiful box in the mail that had these awesome activities that they could not wait to get their hands on. So, I think that it still had some level of excitement”.

#### Attendance

Research staff joined video sessions to log attendance. The majority (73.33%) of treatment families attended at least one *TT STEM* Zoom session, with an average attendance of 39.58% for the eight Zoom sessions. Only two families attended all eight video sessions. The unit introductions had higher attendance (*M* = 46.67%) than the follow-up sessions (*M* = 32.50%). Supplementary Table S5 details attendance by workshop and language. Using separate OLS regressions, we examined if family characteristics predicted attendance: mother/father education, mother/father reported STEM job, household income, and race/ethnicity. We found that higher levels of maternal education were associated with higher attendance (*p* = 0.034). For race, we found that parents who self-identified as Black/African American had lower attendance (*p* = 0.013). For families that did not attend two or more funshop events, most reported reasons were competing priorities of work, childcare for other siblings, or limited time. Two parents reported their children’s lack of interest in the video sessions as the reason for limited participation. No families reported internet or technology barriers.

#### Use of STEM activities

Treatments parents reported in an exit survey on their utilization of the provided activities. Treatment families reported that they utilized most of the provided YouTube read-aloud (85%) and at least two of the three provided activities (85%) in each of the four thematic events. However, we had a low response rate for these parent surveys, which could suggest that about half of families did not utilize the materials, which would bring average utilization down to a low rate of about 39%. More detailed activity usage data for each unit are in Supplementary Table S6.

#### Parent involvement barriers

At the posttest, we asked treatment parents to describe barriers to doing STEM with their children. The most salient barrier to parent involvement was *Limited Time* (*n* = 11 of 20 respondents, 55%). This theme was exemplified by responses such as “Time and energy. Our busy schedules require so much from parents, but this was a nice reminder that many everyday activities can be science and math activities too.” Several parents who noted limited time was a barrier also said that the program helped them later STEM learning into their existing family routines. For example, one parent listed “Time to organize and plan” was a barrier, but said until this program she was “unaware of simple ideas and ways to be creative with objects around the house. I think these things come naturally to educators, but not everyone.” The second part of her response was further coded for the barrier of limited *Information/Knowledge* (*n* = 6, 30%) that included similar parent barriers such as “not knowing what type of projects to do with a child. Receiving ideas was awesome and helpful.” Additionally, five parents (25%) said lack of *Resources/Materials* was a barrier to doing science and math activities at home, saying their challenges were as follows: “Availability of material” or “Ideas, supplies.” Despite these barriers, several parents learned that specialized STEM materials were not the only way to promote learning, saying, “Realizing that parents do not need to buy additional materials. Using what is available like [counting] the chairs in the house or cereal bits to count”.

### RQ2: parent involvement

Contrary to our expectations, there were no main effects on parent involvement in STEM from the quantitative Likert scale survey asking how often families did various types of STEM activities in a typical week—see Table 4. The effect size (ES) was small (*ES* = 0.18, *p* = 0.618). However, qualitative analysis of parent text messages and posttest surveys indicated that treatment parents reported various new ideas, goals, and ways they were supporting their child’s STEM learning.

**Table 4:** Main impact models comparing treatment group to control condition.

	Model 1				Model 2			
	ITT	Standard Error	*p*-value	ES	ITT	Standard Error	*p*-value	ES
Parent involvement								
Treatment	0.08	0.14	0.573	0.16	0.09	0.18	0.618	0.18
Child science interest								
Treatment	−0.02	0.10	0.816	−0.05	−0.09	0.11	0.456	−0.19

The control condition is the reference value (0), and treatment is set to 1. Treatment refers to the virtual workshops and TT STEM program. Basic controls refer to the language of the measure and the child’s age in months. Child demographic characteristics refer to the child’s gender, race, ethnicity, and highest education from a caregiver.ES = effect size.+*p* < 0.10; *p < 0.05; **p < 0.01; ***p < 0.001.

The most prominent strategy, reported by treatment parents in 44.9% of responses (*n* = 22), was *Observing/Reasoning* as they collected data or made comparisons with their child—a strategy emphasized in funshop 3 but present in all events/activities. For example, one parent explained how they promoted observing and reasoning during cooking: “Nosotros seguimos instrucciones de una receta para un pan de plátano. Buscamos los ingredientes. Los separamos en seco y mojado. Hablamos en cuanto el procedimiento y vimos el proceso de crecimiento del pan. Y al final lo disfrutamos (English translation: we followed instructions from a recipe for a banana bread. We looked for the ingredients. We separated the dry and wet. We talked about the procedure and saw the process of growing the bread. And in the end we enjoyed it).”

The next most common strategy treatment for parents, reported in 38.8% of responses (*n* = 19), was *Adapting/Extending* the provided STEM activity to promote additional informal learning—an approach emphasized in all funshops. This was exemplified by a response from a parent who adapted and extended the provided color mixing activity by using new materials to promote literacy: “We mixed finger paint colors in shaving cream to make new colors and practiced handwriting in it.” This response was also coded for *Literacy,* which was a minor theme, with 14.3% (*n* = 7) treatment parents reporting that they embedded literacy (writing and reading) in their STEM explorations.

The third most common way parents promoted STEM was *Numeracy* (*n* = 15, 30.6%), which was the focus of the funshop 2. Numeracy was coded in parent statements such as, “We played Monopoly counting money and spaces.” or “[We are] counting more objects around us”.

Multiple parents reported goals related to *Asking Questions/Promoting Curiosity* (*n* = 8, 16.3%)—a theme introduced in funshop 1 and promoted throughout the program. Parents expressed goals, such as “encouraging the engineering spirit by providing materials and letting them create whatever comes to mind.” Another parent mentioned promoting their child’s interest in technical vocabulary, such as *experiment, estimate,* and *exploring.*

A small number of parents (*n* = 5, 10.2%) reported involving their child in *Problem-Solving/Engineering Design* activities—the focus of funshop 4. A sophisticated example included: “We learned about thermodynamics and heat transfer when our fan to our condensing unit stopped operating. We were able to use water and air to remove the heat from the refrigerant as it passed through the compressor. I had him and his sister testing the resistance across a fuse in our HVAC unit”.

Although only 10 of the 30 waitlist control parents provided qualitative responses about their involvement in their child’s STEM learning, the most commonly reported strategy was promoting *Observing/Reasoning* about things in the natural world (*n* = 5); this was also the strategy most frequently reported by the treatment parents. One control parent reported, “We have been going outside in the evenings finding ladybugs and explained how they lived and what they ate.” The second most common theme reported by parents in the control group was *Literacy* (*n* = 4). For example, one control parent said, “(Child name) loves reading so we usually snuggle up and read together. We also made a bookmark and wrote a letter for the teacher, as Teacher’s Day is coming up”.

### RQ3: child outcomes

The primary child outcome was parents’ report of their child’s interest in STEM. No statistically significant effects were detected for parent reports of the child’s general interest in STEM. The effect size was −0.19 (*p* = 0.456); see Table 4. We asked treatment parents to rate their child’s interest in the *TT STEM* activities kits mailed to each family. Parents consistently rated their child’s interest level as “extremely interested” in the *TT STEM* kit activities (*M* = 1.43, SD = 0.14) on a 5-point scale (*1* = extremely interested, *5* = not at all interested).

## Discussion

We evaluated the feasibility and promise of a brief virtual *TT STEM* program focused on four STEM units, which we revised for remote delivery by museum-based STEM educators. Despite positive feedback, there were no significant impacts of this virtual delivery of the *TT STEM* program on the primary survey measures of parent involvement and child STEM interest. However, there were positive qualitative themes demonstrating substantial involvement in informal STEM activities. The most important findings from this research relate to how virtual approaches may increase informal STEM learning convenience and accessibility in some meaningful ways. However, there were salient limitations to the virtual modality that limit the promise of entirely virtual modalities for future STEM family engagement programs.

### Limited virtual impacts on primary parent outcome and shifted responsibilities

Our primary goal was to increase parents’ frequency of engaging their children in home-based STEM learning, as parent involvement is positively linked to student achievement (e.g., Sheldon and Epstein, 2005; Barnett et al., 2020; Ogg and Anthony, 2020). Parents’ qualitative responses indicated that the program showed them how to observe, estimate, explore, and count on their young children. There were small, non-significant increases (ES = 0.18) in parents’ reported frequency of STEM involvement. Although non-significant, effect sizes were similar in magnitude to past, in-person versions of this program (*ES* range − 0.08 to 0.18 at posttest; Zucker et al., 2022). On the one hand, these similar magnitudes of impacts on parent behaviors for in-person and virtual modalities may indicate that both approaches are suitable. However, we conclude that there are two major disadvantages to the virtual modality, discussed below, that suggest it is not currently suitable as a replacement for in-person family engagement events.

There are multiple potential explanations for these null parent findings. It is possible that there were no group differences because parents in both conditions were already rather involved in supporting their child’s science learning at home; however, descriptively, families only reported doing STEM activities two or three times per week. Another explanation is that the virtual delivery was not of sufficient intensity to change parent behaviors. Indeed, potential challenges of virtual approaches are reduced intimacy with the facilitator and reduced social interactions with other families, which promote behavior change (Sullivan and Strawhacker, 2021). This reduced intimacy and interaction with the informal educator and other families in their community is the first shortcoming of the virtual approach. The STEM educators felt less efficacious when facilitating virtually because they could not answer questions and circulate the room to provide support while families did the STEM activities. Moreover, the qualitative responses from treatment parents indicated that limited time to do STEM with their children was the primary barrier to their involvement.

A second problem of the virtual modality is that the burden of facilitating informal STEM learning is largely shifted to parents in the virtual modality. Given that limited time was the primary barrier parents reported to doing informal STEM, it was likely challenging to ask these busy parents to find time to do 12 asynchronous STEM activities with their child. These exact same activities were not perceived as challenging or overwhelming to complete when families used them at prior in-person events and rotated through workstations where facilitators set up and demonstrated activities. For in-person facilitation, STEM educators also circulated the room, providing support and feedback as families completed activities. Indeed, there are more steps for parents to complete one of the four virtual units (i.e., (1) adding the Zoom session and links to the parent/family calendar, (2) logging into Zoom and attending introductory chat, (3) following texted links or QR codes to view instructional/modeling video for the first kit activity, (4) setting up materials for the mailed kit activity [some of which are messy], (5) completing the activity with your child [while reducing or ignoring competing priorities for parent’s attention in their home], (6) repeating steps 3–5 for the next two activities in your kit, and (7) attending the debrief chat). These steps may be spread out over several days or periods of time that are convenient for the family. In contrast, with the in-person modality, parents are largely responsible for simply attending the funshop. There are not only fewer total steps for completing activities in person [i.e., (1) attending the event, (2) listening to instructions/modeling by museum educators, (3) rotating through three to five activity stations, and (4) sharing out or debriefing with other families at the event], but there are also fewer cognitive and memory demands placed on parents when in-person because the facilitator sets up the space and guides participants through activities in one 60–75-min period. Parents must also be more responsive to their child’s desires and motivation to participate in STEM activities when they pick the time to do these at home, whereas in the social context of in-person learning, most young children are eager to rotate through the stations with their parent/caregiver. In sum, the virtual modality reduced intimacy and support with the facilitator and shifted many responsibilities to parents to orchestrate a multi-step process of informal learning in ways that may have run counter to our goal of broadening, feasible access to STEM.

### Limited child impacts for a brief virtual approach

Our primary goal for children was to increase their broad interest in science and math, but there were no significant gains and a negative trend on this outcome (ES = −0.19). Although treatment parents reported high interest for their children during the provided STEM kit activities, this high enjoyment did not transfer to group differences in a more distal parent report of their child’s general interest in science. Given the lack of significant parent outcomes, the lack of impacts for children is not surprising. The limited duration of this brief four-unit program may also explain these null findings, as low-intensity family approaches are unlikely to impact children’s outcomes (Grindal et al., 2016). Other measurement approaches would be more sensitive, such as in-depth parent interviews on children and family’s STEM interests (Pattison et al., 2022) or innovative apps that allow slightly older children to check in during their informal STEM activities to document interest, setting, and engagement (Morris et al., 2019).

### Key lessons learned for virtual family engagement programs

Museum-based STEM educators and other family engagement specialists ask transformative questions about where informal science learning can occur and how to broaden access (e.g., Ishimaru and Bang, 2016; Ash, 2022). This study reimagined a museum outreach program in a virtual modality to consider if it is feasible to remotely deliver the *TT STEM* program to socioeconomically and linguistically diverse families of young children. The primary affordances of virtual learning were high satisfaction with the quality of activities and the convenience of the virtual format for families. Another benefit was broadened geographic access, including some non-local families and a few families traveling with their STEM kits while joining remote sessions. Yet, a major barrier was that the program did not adequately reach subgroups who may have benefited most. That is, parents with lower education levels and Black/African American families were significantly less likely to attend virtual events. Thus, offering virtual options may be convenient but not sufficient for increasing equitable access and the uptake of family education program goals. These findings align with the literature on virtual approaches where typical benefits are convenience, but known challenges are reduced closeness with the facilitator, limited social learning opportunities, and technology barriers (Sullivan and Strawhacker, 2021; Takeuchi et al., 2021).

Regarding attendance, parents attended an average of 39.58% of virtual sessions. This is commensurate with rates of 35–60% attendance in other in-person family education research studies that do not pay parents to attend (e.g., Heath et al., 2018; Kim et al., 2019). Although 85% of responding parents used the provided STEM activities, we had a low response rate for these parent surveys; thus, if we assume a non-response is linked to not utilizing the activities, then just over a third of families would have utilized materials. Thus, we feel cautious in terms of drawing conclusions about how useable this type of virtual STEM program is for families of young children. We tried to alleviate barriers to the uptake of the program. Families could select a synchronous video session at a preferred time and language and could complete the asynchronous hands-on STEM kit activities at a convenient time and place. We texted parents’ tips and links to online extension activities that minimized resource demands using only typical household objects. Yet, in this sample, the majority of parents reported the primary barrier to supporting science at home was time constraints. This virtual program’s flexible scheduling for doing STEM activities did not alleviate these families’ time constraints. For any busy parent with competing demands on their time, and particularly for families experiencing poverty, researchers need to continue to explore innovative, in-person approaches to layering STEM into places families already spend time, such as grocery stores, laundromats, and local parks (Bustamante et al., 2019), as well as innovative virtual approaches (McCarthy et al., 2013; Rosenfeld et al., 2019).

### Limitations and future considerations for virtual replications

The most salient limitations of this study were the narrow set of outcome measures, the modest intensity of only four thematic units facilitated over 10 weeks, and the relatively small sample. This duration may not have provided enough content coverage and time for parent behavior changes and increased child science interest. A second limitation we noted above is that our generalized measure of child STEM interest was based solely on parent reports, not observations. A third limitation is the COVID context. The salient challenges families were facing in balancing parental responsibilities while supporting their child’s learning during the pandemic (Garbe et al., 2020) may have attenuated or skewed our findings. Indeed, this is likely an atypical sample of education-oriented parents who were willing to sign up for a family engagement program during the pandemic; however, this should have been equally skewed across the randomly assigned groups. Another limitation was differential attrition (i.e., higher attrition for the treatment group than waitlist control). We do not have reason to believe that the treatment was overly burdensome for all families, given high satisfaction ratings from parents. However, it was troubling to find great attrition for treatment. We are also troubled by the shifting of various logistical responsibilities from informal STEM educators to parents who had to coordinate many more steps for the virtual than in-person approaches.

There are important sampling and procedural differences to note when comparing the results of this conceptual replication study to the prior study (Zucker et al., 2022). First, in terms of generalizability, the first study recruited entirely from schools that served a majority of students experiencing economic disadvantage. The replicated study recruited a new sample of families from some of these same schools but also added recruitment via social media because of low initial enrollment. This resulted in a current sample that was more socioeconomically diverse than our initial study. Second, although we drew materials and procedures from the same TT STEM program as the initial study, there are inherent differences in the approach that is appropriate for virtual facilitation compared to in-person programs. We detail these differences in supplemental materials (Supplementary Tables S7, S8) that also include a checklist for how we organized the virtual procedures, which may be of interest to others considering hybrid family engagement models.

## Conclusion

In sum, high-quality, virtual STEM family engagement approaches may be feasible, yet our initial findings do *not* suggest that offering virtual events alone can effectively disrupt inequitable access to STEM family engagement in ways that make meaningful impacts on parent involvement and child science interest. Thus, we conclude that future iterations of *TT STEM* should avoid entirely virtual modalities. We may include both virtual and in-person formats in future programs so families can choose what works for them. We encourage other educators to consider experimentation with hybrid options across a broader student age span while considering issues of digital equity and appropriate cultural and linguistic approaches for diverse families to help ignite their child’s interest in STEM.

## Data availability statement

The raw data supporting the conclusions of this article will be made available by the authors upon request.

## Ethics statement

The studies involving humans were approved by Committee for the Protection of Human Subjects at the University of Texas Health Science Center at Houston's McGovern Medical School. The studies were conducted in accordance with the local legislation and institutional requirements. Written informed consent for participation in this study was provided by the participants' legal guardians/next of kin. Written informed consent was obtained from the individual(s), and minor(s)' legal guardian/next of kin, for the publication of any potentially identifiable images or data included in this article.

## Author contributions

TZ: Conceptualization, Formal analysis, Funding acquisition, Investigation, Writing – original draft, Writing – review & editing. MM: Formal analysis, Validation, Writing – original draft. MA: Project administration, Supervision, Writing – review & editing. CM: Conceptualization, Funding acquisition, Project administration, Resources, Supervision, Writing – review & editing. DD: Data curation, Writing – review & editing, Investigation.
