# Supplementary material for: Virtual Teaching Together: engaging parents and young children in STEM activities
Source: Front Psychol. 2024 Feb 6;14:1334195. doi: 10.3389/fpsyg.2023.1334195 (PMC10876996; doi:10.3389/fpsyg.2023.1334195)

Supplementary Material

**Virtual Teaching Together: Engaging Parents and Young Children in STEM Activities**

Table SM1

*Teaching Together STEM Virtual Treatment Components*

| **Unit Name, Topic: Description^a^** | **Key STEM Dimensions of Home Activity Kits^b^** | **Sample Text Messages^a^** | |
| --- | --- | --- | --- |
| **1. “What’s the Big Idea”** **STEM Language**: Parents were introduced to the overarching concept that you can increase your child’s curiosity about the world through rich conversations that include asking open-ended questions and routinely explaining technical and scientific vocabulary. | - **Asking questions**: Invent a color / Inventa un color - **Carry out investigations:** Water Drop Art / Arte de Gotas de Agua - **Planning investigations**: Nature Detective/ Detective de la Naturaleza | **Tip:** Teach Big Words - Help [child_first_name] get ready for Kinder by teaching [him_her] big words with simple explanations. For example, say, “When we cook, we use cooking equipment.”  **Extension**: Using big words can help build your child's vocabulary. Follow this link for a fun activity: https:// cliengagefamily.org/sensory-popcorn/ |  |
| **2. “Math Rules!” Early Math**: Parents learned how to integrate counting, number identification, and comparison talk into everyday family activities and informal science activities. | - **Using computational thinking**: Pom – Pom Toss / Lanzamiento de Pom-Pom - **Observing patterns**: Pattern Bracelets / Pulseras de Patrón - **Using mathematics and estimating:** The Right Fit / Encájalos Perfectamente | **Tip:** Count Together - Counting and recognizing numbers are some of the first early math skills children need to learn. Encourage your child to count items during everyday routines! For example, when cleaning up ask your child to pick up a set number of objects and count with [him_her].  **Extension:** Exploring different ways to group and compare objects helps children learn math and language skills. Follow this link for a grouping activity: https:// cliengagefamily.org/super-sort/ |  |
| **3. “Show What You Know”** **Gather Data like a Scientist**: Parents explored how to gather information as you interact with their child to include counting, tallies, and charts as simple forms of data in daily life. | - **Structure and function:** Animal Hatchlings / Cría de Animales - **Communicating information:** Starburst Graph / Gráfica de Starburst - **Structure and function:** Test Flight / Vuelo de Prueba | **Tip:** Gather and Record Data - Collecting data is simple and easy. When you ask "How long" or "How many,” those numbers are data! When doing a daily routine (dishes, bathing, etc.), time yourself and [child_first_name] say, "Let's see how long it takes us to do this." Do this throughout the week and keep track of your data.  **Extension:** By gathering data and understanding findings, you help your child build tools for doing science. Follow this link for a fun activity: https:// cliengagefamily.org/water-and-ice/ |  |
| **4. “Dream it Build it”** **Engineering**: Parents were presented with ways to encourage tinkering and creative problem solving within playful activities and common household materials. | - **Engineering process:** Bridge Builders / Constructores de Puentes - **Engineering design/creativity:** Invent A Tool / Inventa una Herramienta - **Engineering design/tinkering:** Hoop Gliders / Aros Voladores | **Tip:** Take on a Challenge - Encouraging [child_first_name] to look for many ways to solve problems helps [him_her] think like an engineer. Design a solution to a problem at your house, ask "How can we fix this broken object?" or "How can we better organize these materials?"  **Extension:** Challenge your child to think of ways to build a bridge strong enough to hold toy animals. Follow this link for a fun building activity: https:// cliengagefamily.org/build-a-bridge/ |  |
| **^a^** Events and text message components were provided in the parents’ preferred language of English or Spanish.  **^b^** Dimensions are based on NRC (2012) Science and Engineering Practices, Crosscutting Concepts, and Disciplinary Core Ideas. All printed kit materials had bilingual English and Spanish written instructions. | | |  |

Table SM3

Participant Flow Through Research Activities

| Research Activity | Definition and Sample |
| --- | --- |
| Consent and Pretest | We consented 68 families, but only 60 families completed pretest and were deemed part of the sample. |
| Randomization | 60 participants were randomized at the parent-child dyad level - 30 to treatment, 30 to waitlist control. |
|  |  |
| Baseline Equivalence Check | For some characteristics (see Table 3), the magnitude of the non-significant estimated differences as measured in standard deviations from the waitlist control group mean exceeded the threshold of \|.25\| SD, the What Works Clearinghouse for baseline equivalence. For example, there was a larger proportion of Asian children (ES=-0.32) and higher household income (ES=-0.43) in the waitlist control group compared to the treatment group. The treatment group had higher levels of African American children (ES=0.30), parent involvement (ES=0.32) and lower levels of parent self-efficacy (ES=-0.44). However, the overall F-test of baseline equivalence using all of the covariates in Table 3 was not statistically significant, indicating overall balance by treatment status (F(18,20)=1.37, p=0.247). |
| Attrition at Posttest | 50 participants remained in posttest analytic sample. We defined attrition as a participant missing a posttest outcome. From the original 60 participants who were randomized (30 to treatment, 30 to waitlist control), 10 did not have posttest assessment data on child STEM interest or outcomes and parent involvement outcome.   - There were 3 attriters in the waitlist control group (attrition=10%) - There were 7 attriters in the treatment group (attrition=23.33%).   Overall attrition was 16.67% and differential attrition was 13.33% which falls outside the recommended What Works Clearinghouse liberal attrition standards for low threat of bias. |
| Differential Attrition by Demographics | Table SM3 in the online supplemental material presents differences in demographic and family characteristics as well as baseline measures between attriters (*n*=10) and non-attriters (*n*=50). Significant differences were found on mother’s highest level of education where attriters had lower levels of education than non-attriters (*ES*=-.75, *p* = .029). Attriters also had lower levels of parent STEM value than non-attriters (*ES*=-.41, *p* = .018). No other differences were statistically significant. |

| Table SM4 | | | | | |
| --- | --- | --- | --- | --- | --- |
| Differences between Attriters and non-Attriters; Means and (Standard Deviations) | | | | | |
|  | Attriters  (*n* = 10) | non-Attriters  (*n* =50) | Unstandardized regression coefficient (Attriters - non-Attriters) | Difference as Effect Size | *p*-value |
| Demographic and Family Characteristics | | | | | |
| Child Female? | 0.60 (0.52) | 0.46 (0.50) | 0.14 | 0.27 | 0.427 |
| Other language at home? | 0.40 (0.52) | 0.52 (0.50) | -0.12 | -0.24 | 0.497 |
| Mother's highest level of education | 4.80 (2.15) | 6.74 (2.56) | -1.94* | -0.75 | 0.029 |
| Father's highest level of education | 4.90 (2.02) | 5.56 (2.98) | -0.66 | -0.23 | 0.507 |
| Mother STEM-related career | 0.60 (0.52) | 0.34 (0.48) | 0.26 | 0.53 | 0.127 |
| Father STEM-related career | 0.30 (0.48) | 0.60 (0.49) | -0.30+ | -0.60 | 0.084 |
| Is child Hispanic? | 0.40 (0.52) | 0.43 (0.50) | -0.03 | -0.06 | 0.870 |
| Child's race |  |  |  |  |  |
| Black | 0.50 (0.53) | 0.24 (0.43) | 0.26+ | 0.57 | 0.099 |
| White | 0.50 (0.53) | 0.66 (0.48) | -0.16 | -0.33 | 0.346 |
| Asian | 0.0 (0.00) | 0.16 (0.37) | -0.16 | -0.47 | 0.180 |
| Other | 0.10 (0.32) | 0.04 (0.20) | 0.06 | 0.27 | 0.435 |
| Household income | 4.33 (2.00) | 5.21 (2.07) | -0.87 | -0.42 | 0.257 |
| Baseline measures | | | | | |
| Parent involvement^b^ | 2.33 (0.58) | 2.56 (0.51) | -0.23 | -0.44 | 0.212 |
| Child STEM Interest^a^ | 3.28 (0.40) | 3.49 (0.44) | -0.21 | -0.49 | 0.164 |
| *Note.* Attriters are defined as those parents who did not complete a post-survey. Unstandardized regression coefficients were obtained from a regression predicting the characteristics on attriter status, adjusting standard errors for clustering at the classroom-level. +*p* < .10; **p* < .05; ***p* < .01; ****p* < .001  ^a^Ranges from 1=strongly disagree to 4=strongly agree  ^b^Ranges from 1=none to 4 everyday Education was measured as an 8-category variable ranging from 1 to 8 where 1 represents <=8th grade and 10 = master or post-graduate degree. Household income was measured as an 8-category variable ranging from 1 (11K or less) to 8 ($150K or more).  Overall F-test for the posttest sample where all variables listed in table were used to predict attrition was statistically significant, *F*(19, 26) = 0.94, *p* = 0.552. | | | | | |

Table SM 5

Parent and Child Attendance at Virtual Chats/Events

| **Initial Funshop Attendance** | | | | |
| --- | --- | --- | --- | --- |
| **Event Title** | **English**  **(n = 26)** | | **Spanish**  **(n = 4)** | |
|  | Children | Adults | Children | Adults |
|  | *n* | *n* (%) | *n* | *n* (%) |
| Intro to Funshop 1 What's the Big Idea? | 13 | 14 (54%) | 3 | 2 (50%) |
| Intro to Funshop 2 Math Rules! | 11 | 12 (46%) | 4 | 2 (50%) |
| Intro to Funshop 3 Show What You Know | 10 | 9 (35%) | 2 | 1 (25%) |
| Intro to Funshop 4 Dream It, Build It | 15 | 14 (54%) | 3 | 2 (50%) |
| **Follow-up and Sharing Attendance** | | | | |
| **Event Title** | **English**  **(n = 26)** | | **Spanish**  **(n = 4)** | |
|  | Children | Adults | Children | Adults |
|  | n | n (%) | n | n (%) |
| Follow-Up to Funshop 1 What's the Big Idea? | 9 | 7 (27%) | 3 | 1 (25%) |
| Follow-Up to Funshop 2 Math Rules! | 11 | 14 (54%) | 2 | 1 (25%) |
| Follow-Up to Funshop 3 Show What You Know | 3 | 9 (35%) | - | - |
| Follow-Up to Funshop 4 Dream It, Build It | 8 | 10 (38%) | 2 | 1 (25%) |

*Note.* This table summarizes the number of attendees at each Funshop delivered in English and delivered in Spanish. The count of potential attendees is based on caregiver self-reported language preferences. For English Funshops, the attendee count reflects the cumulative sum across two offering dates, as each English session was conducted twice to accommodate additional families indicating an English language preference.

| Table SM6  Parent Reported Activity Completion | | | | | | |
| --- | --- | --- | --- | --- | --- | --- |
| **Related Funshop** | **Survey Respondents**  **(n, detailed below)** | | | **Intervention Sample (Survey respondents + non-respondents, n = 30)** | | |
|  | Completed Survey | Watched Read-aloud/1 | Completed Activities/3 |  | Watched Read-aloud/1 | Completed Activities/3 |
|  | n (%) | Sum, % | Mean (SD), % | n | % | Mean (SD), % |
| Funshop 1 What's the Big Idea? | 14 (47%) | 11, 79% | 2.43 (1.16), 81% | 30 | 37% | 1.13 (1.46), 38% |
| Funshop 2 Math Rules! | 15 (50%) | 15, 100% | 2.50 (.91), 87% | 30 | 40% | 1.30 (1.47), 43% |
| Funshop 3 Show What You Know | 12 (40%) | 9, 75% | 2.50 (1.00), 83% | 30 | 30% | 1.00 (1.39), 33% |
| Funshop 4 Dream It, Build It | 14 (47%) | 12, 86% | 2.71 (.83), 90% | 30 | 40% | 1.27 (1.48), 42% |
| **Total Average** |  | **M = 0.85 (*SD* = 0.36) 85%** | **2.56 (*SD =* 0.96), 85%** |  | **37%** | **1.18 (*SD* = 1.44), 39%** |

Table SM7

Comparison of Original Study and Conceptual Replication

|  | **Original Study (Author et al., 2022)** | **Replication Study** |
| --- | --- | --- |
| Sample | We recruited from schools where 92% of students received free/reduced lunch. This resulted in a sample with median yearly household income of $20,001-$30,000. | We recruited from these same schools, but also via social media. This resulted in a sample with median household income was $40,001-$70,000 and a sizeable range from <$11,000 to >$150,000. |
| Setting | The first study occurred in the 2019-20 school year. The COVID pandemic started during the last set of workshop theme and impacted posttest. | The second study occurred in the 2020-21 school year. The COVID pandemic was ongoing but treatment started in March when things were normalizing (i.e., local schools re-opened in-person in October of that school year with masking). |
| Conditions | We randomly assigned families to business as usual (BAU) control or one of three treatment (Tx) groups A-workshops only, B-workshops plus take-home materials, C-workshops plus take-home materials plus $2.50 reward for doing STEM photo or text. | We randomly assigned families to business as usual (BAU) waitlist control or one treatment (Tx) group. After posttest, we offered families in the BAU control group one virtual sessions, as this improved recruitment and increased access to the STEM experiences. |
| Materials | Six workshop themes offered: (1) “What’s the Big Idea” STEM Language; (2) “Let’s Figure It Out” STEM Inquiry; (3) “Math Rules!” Early Math; (4) “Show What You Know” Gather and Analyze Data; (5) “Dream It, Build It!” Engineering; (6) “Picture This” Systems and Models. | Four of the same workshop themes offered: (1) “What’s the Big Idea” STEM Language; (2) “Math Rules!” Early Math; (4) “Show What You Know” Gather and Analyze Data; (5) “Dream It, Build It!” Engineering. |
|  | Each workshop included three to five activity stations linked to the theme. These materials were setup by the museum STEM educator in the school facility and usually afterschool at a time the teacher/school liaison recommended. | Museum STEM educators mailed bilingual kits with same materials from workshops, but only a portion were selected that were feasible to mail (see SM2). Parents determined where and when to do these activities. |
|  | Nine extra bilingual take-home activity kits that aligned with workshop themes (see SM4 of Author et al., 2022). | No extra take-home activities were used because this sheer volume of materials was high cost and expected to be overwhelming for parents to facilitate. |
| Methods | In-person events were hosted at child’s school library or cafeteria with text message event reminders as well as flyers and stickers that teachers placed on each student’s shirt before events to promote attendance. | Virtual events were organized by time and language preference with text message Zoom links and reminders sent to parents. Flyers were also sent home by teachers in participating schools with all possible time offerings. |
|  | Text messages were sent after each workshop with tips and online extension activities. | Text messages were sent after each virtual introduction that used the exact same tips and online extension activities as the initial study. |
| Measures | Parent involvement in STEM pretest/posttest rating with items that ask “In the past week, number of times you…” talked to your child about shapes, weather, nature, etc. | We used the same pretest/posttest parent involvement in STEM scale as the original study. |
|  | No child interest measure was used, but was suggested by journal/peer reviewers. | Added parent report of child’s general interest in STEM at pretest and posttest. |

Table SM8

Checklist for Successful Virtual and Hybrid Informal STEM Family Engagement

| **Component** | **Key Activities** |
| --- | --- |
| Marketing | - Advertise with flyers that explain the virtual or hybrid approach - Ensure families provide address for mailing kits that matches where they will be located during the upcoming events - Offer multiple virtual event times - If possible, offer virtual events in common home languages - Send reminders to parents one week and one day before event - Test the link to videoconference events work - Add QR codes for the videoconference link to flyers to improve ease of access |
| Virtual Events | - Welcome families as they join with warm tone - Consider having a welcome slide/music with norms listed (e.g., mute unless speaking, use chat, avoid driving during event) - Encourage brief family introductions - Consider a quick icebreaker activity to immediately engage families - Introduce your content/theme with visual aids such a video or slides - Encourage participants to share their ideas or learning during the event to encourage social participation - Preview the asynchronous activities to build excitement and explain where to find more detailed instructions - Explain how families can contact the ISE with questions as they start the activities |
| Activity Kits | - Consider costs of shipping materials and budget - Plan to ship kits to arrive within 3-7 days before event - Add instructions to wait to open activities until the video chat event is completed so that educators can introduce the theme and build excitement before starting kit activities - Provide detailed, bilingual instructions in the kit with step-by-step photos, if needed - Consider adding links and QR codes to online videos that demonstrate and model activities when done by ISE and families to reduce literacy demands and provide an expert model - Encourage families to post photos and videos doing the activities with a tag that links to the museum or organization’s social media |

Figure SM1. Logic model for virtual Teaching Together STEM program improving adult and child outcomes.


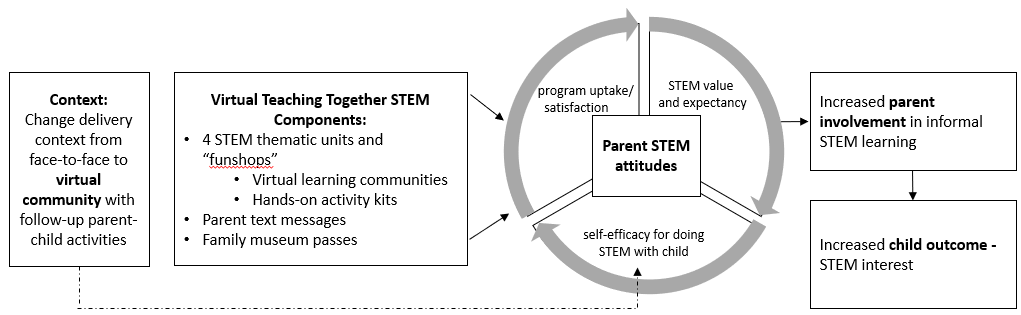

Supplement: Supplementary file 1 [file Data_Sheet_1.docx]
